# Lessons from Peru to reduce under-5 mortality: understanding program implementation and context

**DOI:** 10.1186/s12887-023-03890-w

**Published:** 2024-02-28

**Authors:** Patricia J. García, Anna Larson Williams, Marco H. Carcamo, Amelia VanderZanden, Agnes Binagwaho

**Affiliations:** 1 School of Public Health, Cayetano Heredia University (UPCH), Lima, Peru; 2 University of Global Health Equity, Kigali, Rwanda

**Keywords:** Under-5 mortality, Maternal and child health, Implementation research

## Abstract

**Background:**

Health policymakers aiming to reduce under-5 mortality (U5M) often lack data regarding how successful interventions in other countries were implemented. The Exemplars in U5M Study identified countries that achieved significant reductions in amenable U5M. This case study in Peru used implementation research to explore the contextual factors and strategies that contributed to the successful implementation of key evidence-based interventions (EBIs).

**Methods:**

This research utilized a hybrid implementation research framework and a mixed-methods approach to understand the factors associated with EBI implementation and the successful reduction of U5M between 2000–2015. A desk review of existing literature on EBIs and U5M in Peru was completed, and in-depth interviews were performed with key Peruvian informants to understand the implementation strategies employed and the contextual factors that facilitated or were barriers to success. For the purposes of this analysis, three EBIs were selected and evaluated: antenatal care visits (ANC), facility-based deliveries, and infant vaccination.

**Results:**

Between 2000–2015, the percent of mothers attending at least four antenatal care visits rose from 69% to 96.9%, and the percent of facility-based deliveries increased from 56 to 91%. Three doses of the tetanus/diphtheria/pertussis vaccine, widely acknowledged as a key global health indicator, reached 90% by 2015. Key informants noted that economic growth, financial reforms, strong national commitment to reduce poverty in Peru, and national prioritization of maternal and child health, were important contextual factors that contributed to the successful reduction of U5M. They noted key strategies that helped achieve success during the implementation of EBIs, including utilization of data for decision-making, adaptation driven by cultural sensitivity to address gaps in coverage, and a focus on equity and anti-poverty initiatives with the participation of government, civil society, and political parties to assure continuity of policies.

**Conclusion:**

Several EBIs contributed to the successful reduction of U5M in Peru between 2000–2015. Strategies such as the focus on equity throughout the study period contributed to an increase in coverage of EBIs like ANC visits, facility-based deliveries and infant vaccination which worked to reduce U5M. Understanding how Peru successfully implemented programs that reduced preventable infant and child deaths could be useful to replicating this substantial public health success in other low- and middle-income countries.

## Introduction

Under-5 mortality (U5M) poses a significant global health challenge and is a key indicator for countries aiming to improve maternal and child health (MCH) [1, 2]. Identifying evidence-based interventions (EBIs) that work to reduce U5M is critical [3]. Health policymakers aiming to replicate EBI successes, often lack data regarding how interventions were implemented and the specific strategies that facilitated program success, and there is often a lag between the dissemination of EBIs and their implementation.

Implementation research, the study of how strategies are used to adopt and integrate EBIs into real-world settings to improve individual outcomes and population health, is an important tool in understanding successes and challenges of EBI implementation in low-and middle-income countries (LMICs) [4–6]. However, much of the literature on EBIs in MCH is focused on effectiveness and coverage, without exploring what was done (implementation strategies), and what worked and why (such as barriers and facilitators that can influence choice of strategy and success). The University of Global Health Equity (UGHE) collaborated with the Bill and Melinda Gates Foundation and Gates Ventures for the Exemplars in U5M Study to identify countries that have achieved significant reductions in amenable U5M – deaths preventable through health system-delivered EBIs – between 2000–2015, relative to comparable countries geographically or economically and using implementation research, understand the implementation strategies and contextual factors that contributed to the success of EBI implementation, and identify lessons that could be adapted to other countries.

One of the countries selected as an “exemplar” was Peru. Peru is the third largest country in South America, and had a population of 30.5 million in 2015 [7]. Peru’s U5M dropped from 38.6 deaths/1,000 live births in 2000 to 16.6/1,000 live births in 2015, a 57% decline [8]. The country also achieved similar impressive reductions in neonatal mortality (NM), dropping 51%, from 15.5 deaths/1,000 live births in 2000 to 7.6/ 1,000 live births in 2015 [8]. Although regional disparities remained, suggesting that more work needs to be done, these reductions in U5M occurred across all wealth quintiles, and the gap between quintiles narrowed for both U5M and NM [9–13].

To achieve these dramatic reductions in U5M and NM, health leaders implemented EBIs targeted at preventing and curing common causes of child deaths in Peru. This study explored what is often missing from available published or gray literature: the key lessons in how policies and EBIs were implemented, adapted, and sustained by a country. To achieve this aim, we utilized a hybrid implementation research framework to understand the strategies used to implement and sustain the EBIs, and the role contextual factors played in facilitating or hindering the successful reduction of U5M.

## Methods

This research was a collaboration between UGHE (Rwanda) and the School of Public Health from Cayetano Heredia University (UPCH, Peru) and was part of the multi-country case study, “Exemplars in U5M “[14]. We used a hybrid implementation research framework built from existing frameworks and a mixed-methods approach, described in detail elsewhere [15]. This framework, modified from Aarons and colleagues [16], focused on specific stages of implementation of MCH programs: Exploration, Preparation, Implementation, Adaptation, and Sustainability (EPIAS) [15]. It included contextual factors, which were expanded from the Consolidated Framework for Implementation Research and other research focused on U5M, to reflect factors associated with implementation in LMICs [15–18]. Contextual factors were explored because they are critical to understanding potential success of EBIs.

### Selection of EBIs

When selecting the EBIs for the study we identified, based upon a thorough review of existing literature and guidelines from Millennium Development Goal (MDG) efforts, EBIs known to impact common causes of U5M among infants and children in LMICs. A full list of EBIs identified has been published previously [15]. For this analysis we included those EBIs that were either ongoing or first introduced in Peru during the study period and were more often mentioned by the interviewees.

### Data collection

#### Desk review

A desk review of existing literature on the rates and progress of U5M, policies, strategies and EBIs available as well as the uptake and implementation of these EBIs was performed between January and April 2019 through MEDLINE (PubMed) and Google Scholar using the search terms “child mortality” or “under-5 mortality” and “Peru.” Further searches included specific EBIs, causes of death, or contextual factors as search terms (e.g. “insecticide-treated nets,” “malaria,” or “community health workers”). Articles were reviewed if they included one of the EBIs defined from the list in Appendix 1: Tables 2 and 3, and had qualitative or quantitative information on contextual factors, implementation strategies, or implementation outcomes, and was specific to Peru. This was an iterative process as additional sources (published articles, reports, case studies) were identified and as the information from the key informant interviews were analyzed. The review included health interventions related to amendable causes of death from the neonatal period to early childhood. Notably, interventions that contributed to U5M reduction beyond health-related EBIs were considered out of scope for the desk review but were included as contextual factors. Contextual factors including broader interventions that contributed to U5M reduction such as education, poverty reduction, water and sanitation, and programs designed to improve nutritional status beyond severe acute malnutrition and breastfeeding were also explored. Data from Peru’s Demographic Health Surveys (DHS) [11, 12], published reports, and gray literature on U5M were incorporated to supplement the review. Researchers incorporated additional analyses from the International Center for Equity in Health (Federal University of Pelotas) and the Institute for Health Metrics and Evaluation (University of Washington) to evaluate mortality from an equity lens [8, 13].

#### Key informant interviews

To complement and explore the results of the literature review and quantitative data analysis, we completed in-depth interviews from July to September 2019 with 16 Peruvian key informants exploring the implementation strategies for the different EBIs, as well as the contextual factors that hindered or facilitated program or implementation success. The implementation strategies and contextual factors explored were developed a priori based upon the frameworks used to develop this research [15]. Key informants were selected via a convenience sampling methodology, based upon their participation and experience in the EBIs in this analysis. Key informants included current and former Ministry of Health (MOH) employees responsible for high-level strategic direction of the Ministry; experts in specific disease or intervention areas; implementing partners and other multilateral organizations; donor organizations and non-governmental organizations who had managed partner-supported or partner-led activities; and health researchers who were involved in MCH program implementation between 2000–2015. Some informants represented more than one area or role based on their experience over the 16 years and were interviewed for each of their multiple viewpoints. The total number of key informants included in this study was not designed to reach saturation but instead was intended to cover a breadth of EBIs and implementation strategies. The interviews were conducted and recorded in Spanish, transcribed, and then translated into English.

## Data analysis

Researchers trained in qualitative analysis coded the interviews by organizing ideas into pre-determined themes following the implementation research framework. We used descriptive statistics to determine EBI coverage nationally and subnationally. We combined the primary qualitative data and secondary data using an explanatory mixed-methods approach. We further identified transferable lessons with the potential to be adopted or adapted for other countries that are working to accelerate the decrease in U5M. The primary findings from the desk review and DHS data were combined with the results of the key informants’ interviews to understand better the processes and link implementation strategies, contextual factors, and coverage results.

### Ethical considerations

The study and instruments were reviewed and approved by the Institutional Review Board of Cayetano Heredia University (104276) before the study was performed. The overall project was reviewed by the Rwanda National Ethics Committee, the University of Global Health Equity and Northwestern University and determined to be non-human subject’s research.

## Results

The success in dropping U5M in Peru represented a combination of implementation strategies to improve coverage of EBIs aimed at increasing access to care and reducing disease incidence, as well as contextual factors which enabled this work and influenced overall U5M. Two of the leading causes of U5M in Peru in 2000 were lower respiratory infections (LRIs) (19.3% of all deaths of children under 5 years of age) and diarrheal diseases (5.3%); by 2015, U5M by these causes decreased to 12.2% and 3.2%, respectively [8]. The three leading causes of neonatal death were the same in 2000 and 2015 but with considerable declines: preterm births (6843 per 100,000 in 2000 to 3365/100,000 in 2015), neonatal encephalopathy due to birth asphyxia and trauma (4276/100,000 to 2762/100,000), and neonatal sepsis and other neonatal infections (3134/100,000 to 2724/100,000) [19].

**Fig. 1:**
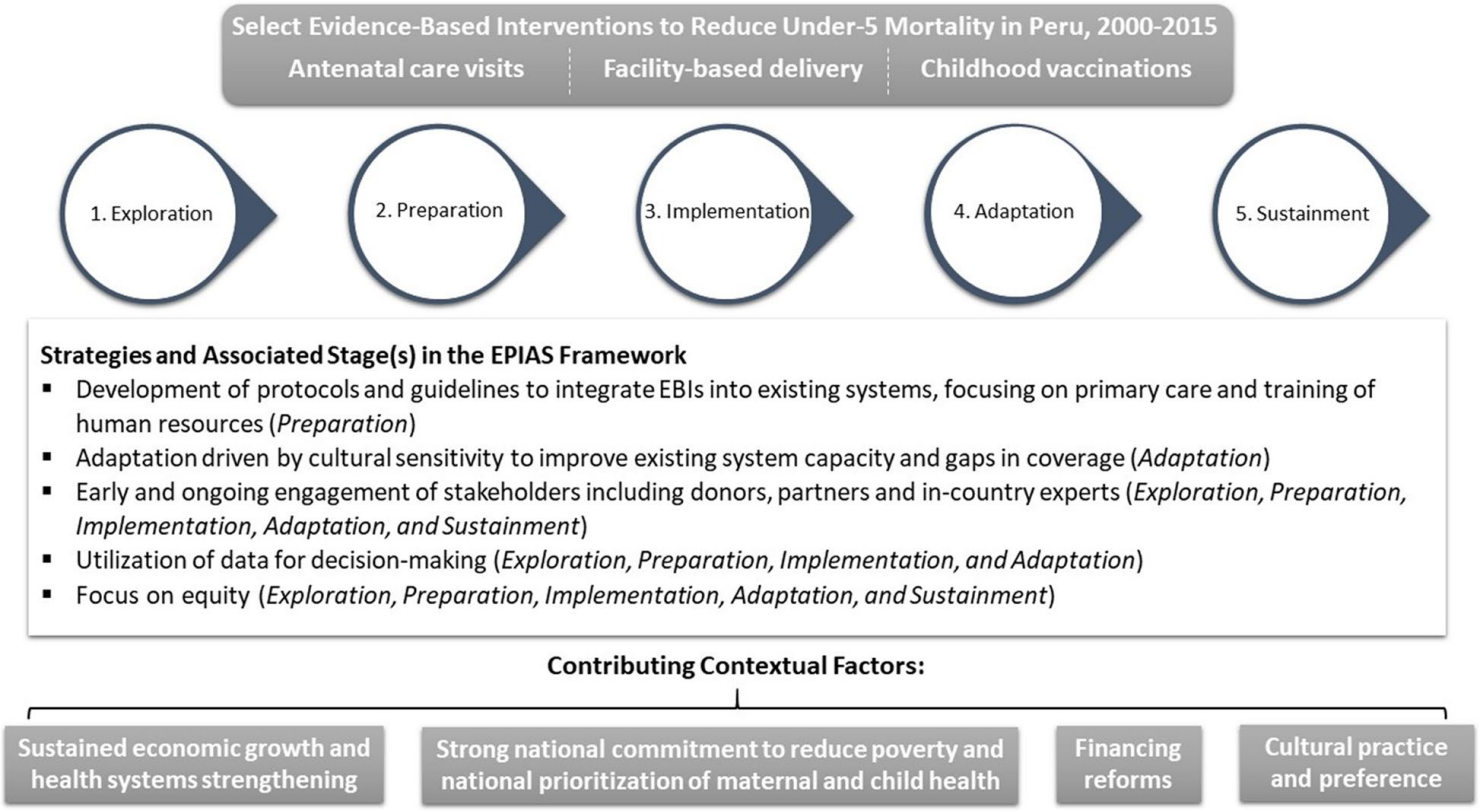
Examples of EBIs, strategies and associated EPIAS stages, and contextual factors

Peru implemented many key preventive and curative EBIs known to address the leading as well as other amenable causes of death for children under 5 years old. While there were several EBIs used during this period [20], in this paper we focused on the most commonly mentioned by stakeholders during the interviews: antenatal care visits (ANC), facility-based deliveries (FBDs), and vaccinations (Fig. 1).

### Overview of the three EBIs

#### ANC visits

**Fig. 2:**
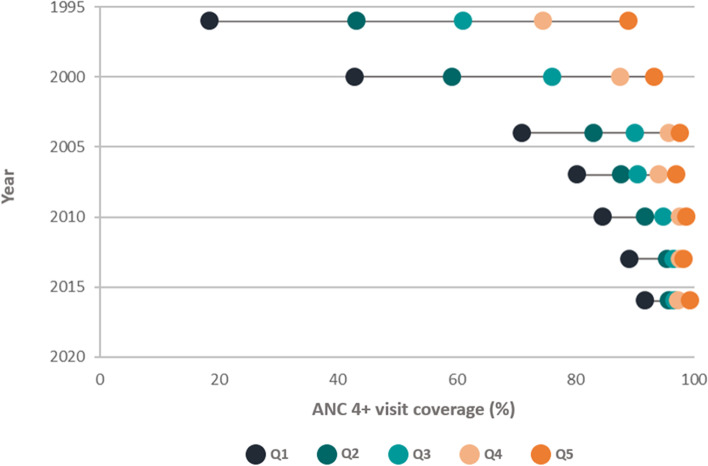
Coverage of four or more antenatal care (ANC 4 +) visits in Peru by wealth quintile, from 1996 to 2016

In 2004, Peru published guidelines recommending that women attend at least six ANC visits [21]. Peru achieved dramatic improvements in the provision of four or more ANC visits (ANC4 +), with an increase in coverage from 68.5% in 2000 to 95.6% in 2015 [12, 22]. The program showed significant declines in the gap in coverage among wealth quintiles (Fig. 2) although some gaps remained in urban and rural areas (96.5% vs 93.2% respectively) in 2012 [12].

To achieve this success, policymakers and program implementers focused on addressing equity gaps in coverage. The ANC4 + visits program was incorporated into a national program which distributed direct conditional cash transfers to low-income families through the JUNTOS program (JUNTOS: National Program of Direct Support to the Poorest https://www.gob.pe/juntos) and into the Comprehensive National Health Insurance System (known as Seguro Integral de Salud in Spanish, or “SIS”). SIS covered services for ANC at public health establishments, including laboratory screenings, two ultrasounds and iron supplementation, and JUNTOS requires (among other activities) for pregnant women to access ANC to receive the benefit of the program (cash transfer), which subsequently increased demand, utilization, and coverage of qualifying health services. [23, 24]

#### Facility-based delivery (FBD)

**Fig. 3:**
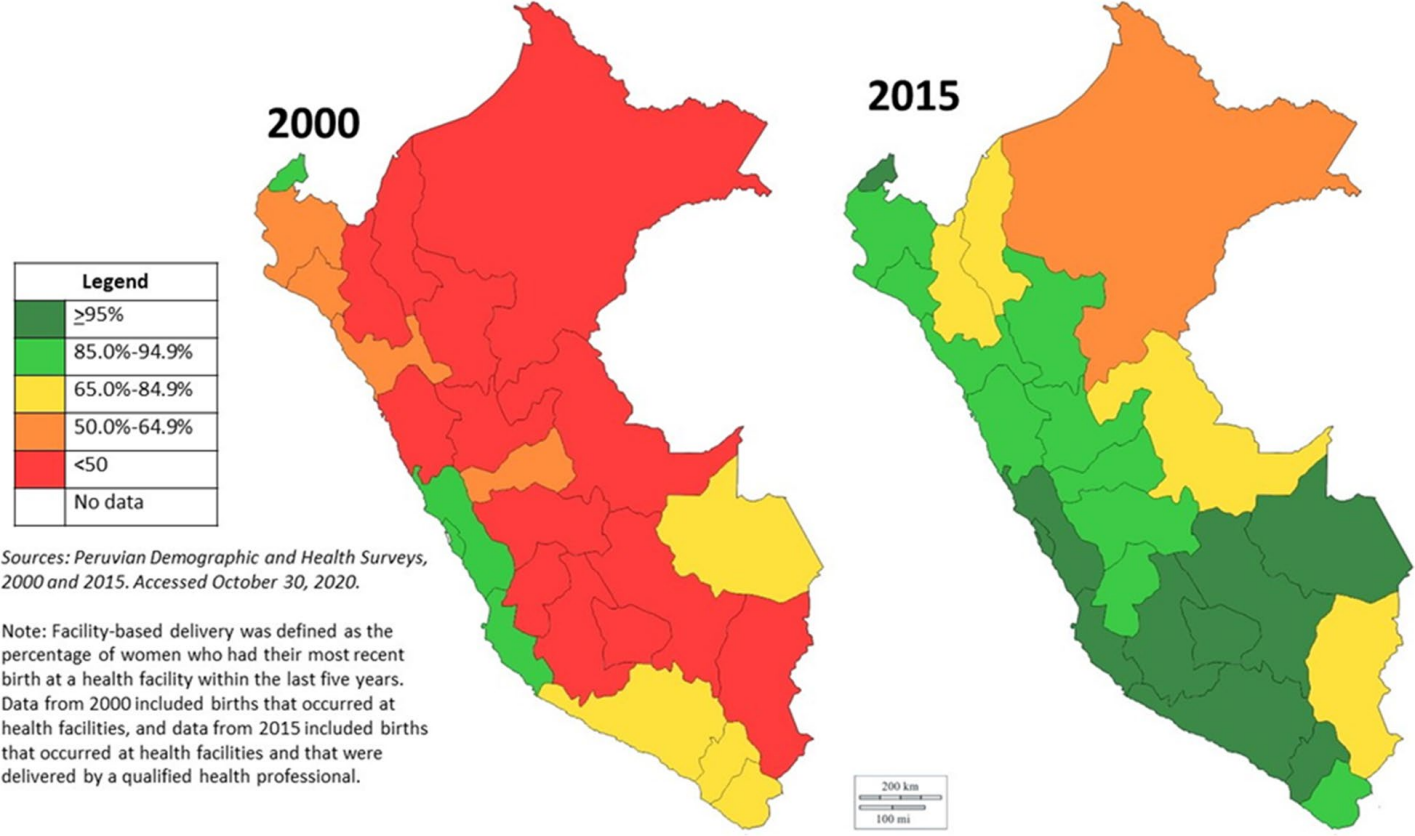
Facility-based delivery coverage by region, 2000 and 2015

In 2000, just over half of deliveries (57.9%) occurred in health facilities, with considerable variation in coverage by department, urban versus rural areas, number of ANC visits, and level of women’s education [11]. Regional differences in coverage rates between 2000 and 2015 are shown in Fig. 3. In the late 1990s, great efforts and resources were dedicated to improve FBD rates, among them Proyecto 2000, which was a collaboration between the Peruvian government and USAID that aimed to improve the quality of maternal and child health services in Peru, including obstetric care at health facilities [25, 26]. By 2015, 91% of all births occurred in health facilities [12]. While FBDs have been sustained over time and reached 92.4% in 2019 [27], disparities remain between lowest-income women and those in other wealth quintiles [13].

#### Vaccinations

**Fig. 4:**
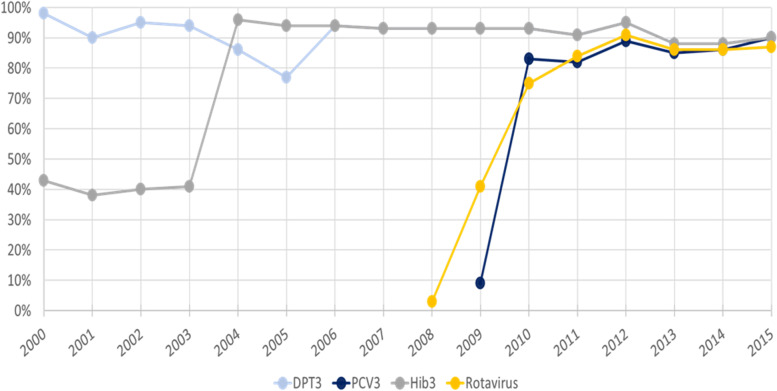
Coverage of select childhood vaccinations in Peru, 2000–2015 DPT3: Completed dose series (3) of diphtheria, tetanus, and pertussis vaccine among 1-year-olds (%). PCV3: Completed dose series (3) of pneumococcal conjugate vaccine among 1-year-olds (%). First introduced in 2008 and scaled nationally in 2009. No coverage data was available for 2008. Hib3: Completed dose series (3) of Haemophilus influenzae type B vaccine among 1-year-olds (%). Rotavirus: Completed dose series (2) of rotavirus vaccine among 1-year-olds (%). First introduced in Peru in 2008 and scaled nationally in 2009. Note: The pentavalent vaccine, which provides protection against hepatitis B, diphtheria, tetanus, pertussis, and Haemophilus influenza type B, was introduced in 2007, which is why the coverage rates for DPT3 and PCV are recorded as the same after that date

Peru achieved high coverage of many pediatric vaccines by 2015, such as the rotavirus vaccine (87% coverage), Hemophilus influenzae type b (Hib) vaccine (90%), three doses of pneumococcal conjugate vaccine (PCV) (90%), and three doses of diphtheria/pertussis/tetanus (90%) (Fig. 4) [28–31]. In the case of PCV, one key informant noted that introduction of the vaccine in a priority area allowed the government to build evidence and gain support for implementation at a national scale.

### Strategies used

#### Adaptation driven by cultural sensitivity to reduce gaps in coverage

Peru utilized culturally-relevant strategies to improve institutional delivery during the study period [32, 33]. The implementation of strategies based on culturally-relevant birthing practices such as “vertical delivery” and launching of maternal “waiting homes”, close to health facilities and similar to typical indigenous houses, are key examples of policies that increased FBDs, especially in rural areas [25]. Research guided the adaptation and was followed by clear national guidelines and extensive training of health providers. One key informant mentioned that this “*allowed mothers to give birth vertically; this is what was done historically, pre-Hispanic, and is physiologically better and right*” (Interview 4, multilateral organization). The incorporation of culturally-relevant practices into EBIs likely contributed to the reduction in deaths through improved use of health facilities and maternal care services [9].

#### Early and ongoing engagement of multiple stakeholders including donors, partners, and in-country experts

For the different strategies, involvement of stakeholders throughout all the stages of implementation was a key step in the process. Technical and financial support was leveraged to aid the feasibility of the introduction and widespread implementation of EBIs.

For example, efforts to increase FBDs among rural women were undertaken in coordination with partners including UNICEF and USAID, which helped in the establishment of the first maternal waiting homes. The Ministry of Health worked on scaling this strategy once the concept was proven, and communities demonstrated their acceptability.

#### Utilization of research and data for decision-making

Data such as those generated from research and from the Peruvian demographic and health survey (DHS known as ENDES in Spanish) and the results-based budget have been utilized by the Peruvian MOH to make high-level policy decisions. The DHS survey data allowed health officials to monitor both key health outcomes and access to the programs that attempted to address those problems. Data was used for decisions regarding implementation of strategies as well as to monitor progress and to make changes when it was deemed necessary. According to one key informant, *“tools like [the DHS survey] allowed us to discuss the quality of the policy. For example, we have seen resource allocation, if we allocate resources for the right programs, we have also been able to see what was going on with rural deliveries that had one of the most complicated areas of coverage”* (Interview 6, non-governmental civil society organization).

Other examples, related to vaccine introduction of rotavirus vaccine and PCV were mentioned by interviewees. For PCV, data was used to help with prioritization, in identifying areas of the country with the highest burden of pneumonia that were also the poorest. For rotavirus, data was used to understand the disease burden and the appropriateness of the intervention through review of existing reports and national data, prior to the introduction of the rotavirus vaccine.

Furthermore, research was mentioned as an important source of data—and not only quantitative data- for policy-decisions. Formative research was key to understand the low numbers facility-based deliveries in the rural areas and to propose and implement the strategies of vertical delivery and maternal “waiting homes”.

#### Development of protocols and guidelines to integrate EBIs into existing systems, with participation of the providers, focusing on primary care and on training of human resources

Nationally-led development and implementation of protocols and guidelines, as well as high-level policies (such as ministerial decrees) accompanied the implementation of key EBIs. Those guidelines and protocols were used to train health providers using participatory strategies, to effectively insert the new activities into existing systems, and to monitor their progress. For example, the MOH developed a protocol for maternal and newborn health that included guidance for at least six ANC visits for pregnant women [21] – which was beyond the WHO minimum guidance of four. “*While international evidence showed a four-visit schedule could be effective, the decision to maintain a six-visit schedule was done in consultation with health professionals across all regions. A very intensive training to understand and implement the guidelines was critical*” (Interview 5, former Ministry of Health employee).

#### Focus on equity

During the study period, Peru developed many national anti-poverty initiatives that targeted equity and made MCH a national and regional priority [9]. For example, the Peruvian government implemented an equity-based approach offering vaccines at health establishments across the country with catch-up campaigns visiting households. When new vaccines like PCV, rotavirus, and Hib were introduced, economically disadvantaged areas of the country were prioritized before national scale-up to ensure coverage in those areas where they were most needed [34]. 

### Contextual factors that contributed to or hindered program success

Contextual factors influenced both EBI implementation and U5M reduction by directly addressing amenable causes of death through increasing prevention and access to care. Peru enacted various policies and initiatives outside the healthcare system broadly impacting the incidence of disease and improving the resiliency of children. One successful effort was the program to reduce stunting in Peru [35], which provided an important context to the reduction of U5M. Key informants noted that economic growth, consistent national-level leadership in MCH and a commitment to anti-poverty initiatives, and health sector financing reforms were significant contextual factors that contributed to the successful reduction of U5M in Peru. Barriers that might have impacted program success included cultural practices and preferences.

#### Sustained economic growth (facilitator)

Despite persisting political and economic challenges, by 2000, the country resolved many of its political issues, and a period of political stability and economic growth began [36–39]. Peru’s gross domestic product grew about 6.3% from 2002 to 2010, and the poverty rate fell from 48.6% in 2004 to 21.8% in 2015 [40, 41]. Peru also increased national health financing, and from 2000 to 2011, total health expenditure per capita more than tripled [42].

Many key informants attributed the reduction in U5M to the macroeconomic policies and economic success of the study period. One key informant went so far as to say, *“There is almost a direct link between economic growth and reduced child mortality”* (Interview 2, public health technical expert)*.* Another informant mentioned: “*It is no secret that if a country recovers financially, it has fewer diseases, the population becomes sick less often, and people can be better nourished…the country’s continued economic growth is an extremely important foundation”* (Interview 16, implementing partner).

#### Strong national commitment to reduce poverty in Peru and national prioritization of MCH (facilitator)

Leadership at the national level prioritized MCH programs through national strategies and policies during the study period. For example, in 2002, the Peruvian government established the National Agreement (known as the Acuerdo Nacional in Spanish, or “AN”), which convened political parties and civil society with the aim to strengthen democracy by promoting equity and social justice, including specific goals to reduce maternal and child mortality and ensure equitable.

access for evidence-based MCH programs [43]. Similarly, the Roundtable for the Fight against Poverty (known as Mesa de Concertación de la Lucha Contra la Pobreza in Spanish, or “MCLCP”) was created in 2001 and aimed to promote dialogue between the government and civil society and utilize a human rights-based approach to address issues of systemic inequality and social exclusion [44].

The MCLCP and the AN were responsible for creating governing agreements and calls upon political candidates to pledge a commitment to them, many of which have been explicit in designating resources to reducing NM and chronic child malnutrition. Through this work, Peru developed a social policy framework to reduce inequities in the groups most affected by poverty, such as indigenous and rural populations. The MCLCP’s strong presence in national politics, and the political will generated from the AN, both contributed to the development of policies and subsequent programs that addressed social inequities, several of which specifically targeted improvement of MCH. One key informant noted that the AN aided in political decision-making by helping national leaders develop a shared vision, saying, “*political decisions have a connotation in terms of deciding what should happen, but after that decision, there has to be a way to make it happen…I think there the national agreement was key to having national goals”* (Interview 4, multilateral organization).

#### Financing reforms (facilitator)

The Ministry of Economy and Finance passed a law in 2007 implementing a results-based budget financial reform and required Ministry-funded programs to adhere to specific and quantifiable results [45]. The performance and results-based indicators are continually monitored and must be measurable through the annual DHS survey. One key informant noted,

“*A major factor that has contributed to the reduction of malnutrition and child mortality has been to implement this resource alignment strategy to budget for effective interventions that lead to a clear outcome. The results-based budget* (PpR acronym in Spanish), *aligns institutions and services, and establishes a series of elements that help you spend what needs to be spent, concentrating the resources on what we know is effective, and not wasting resources on other things”* (Interview 7, government employee).

The results-based budget is a program that monitors outcomes based on evidence, allowing health officials to evaluate the country’s progress toward achieving key MCH indicators. This same key informant commented on the importance of this program as it pertains to child health, adding,

“*The idea was to identify which are the most effective interventions, and allocate resources to these most effective interventions, and from that year you can see how much the country is investing in all children being vaccinated, to avoid mortality, and morbidity, how many children are on target for growth and development”* (Interview 7, government employee).

#### The lack of culturally appropriate services (barrier)

In a diverse multicultural country such as Peru, there is a need to have health services capable of adapting and operating within the local settings. In the case of FBDs, the national coverage in 2000 was nearly 57.9%, but there were wide regional disparities in coverage. For example, coverage was as high as 92.5% in Ica (a coastal urban region), but below 30% in several Andean, jungle, and other more rural regions [11]. Local research results showed the need to develop culturally-based adaptation of maternity services which was started by the Ministry of Health in the early 2000’s. These included new delivery standards, allowing for vertical delivery, accompaniment of family members and traditional midwives during delivery, and increased temperature in health facilities. While regional differences continue, there have been substantial gains in coverage across regions; by 2015, for example, the lowest coverage of institutional delivery at any region in Peru was 64.1% [12].

### Remaining challenges

Although Peru utilized the strategy of focusing on equity, the country has continued to face challenges in achieving equity of coverage of key EBIs. For example, FBDs varied between rural and urban areas and richer and poorer regions, from a high of 99.1% in Callao in 2015 to a low of 64.1% in Loreto (in the jungle of Peru) [12]. This inequity could be explained at least in part by the political and administrative centralization of resources in the capital and urban cities and differential development and access to healthcare services by region and ethnicity [46, 47]. Although the decentralization process in the country officially began on July 28, 2002, even today there are challenges in its implementation, due to lack of regional technical capabilities and resources.

Additionally, while data use for decision-making was an important strategy for the implementation of many EBIs in Peru, there have been ongoing challenges. High-quality, standardized data across all areas of the health system have been challenging to collect in Peru’s highly fragmented health system, and it has been difficult to monitor coverage across all regions or quality of services across all schemes of care.

## Discussion

From 2000–2015, Peru was able to achieve significant progress in coverage of key EBIs including programs to achieve improvements in ANC, an increased rate of FBDs, and high coverage of childhood vaccinations. Key informants noted several contextual factors that contributed to success, including sustained economic growth and health system investment, strong national commitment to reduce poverty in Peru, national prioritization of MCH, and financial reforms. They also noted that adapting programs to be culturally appropriate, early and ongoing engagement with stakeholders, utilization of data for decision-making, developing protocols and guidelines to integrate EBIs into existing systems and train health personnel, and a focus on equity were key strategies that helped achieve success during the implementation of EBIs to reduce U5M. The anti-poverty agenda in Peru not only addressed critical inequities to reduce health disparities between wealth quintiles, but also positively influenced the EBIs and facilitated sustainability in many MCH programs. Prioritizing MCH initiatives at the national level and cultivating political will created a down-stream ripple effect which further facilitated program successes.

These implementation strategies and contextual factors have also supported effective implementation of similar health programs in other contexts. Among others, key contextual factors such as urbanization and improved socioeconomic conditions were attributed to achievements in child health in Iran [48]. In Ethiopia, national programs to reduce poverty, an increase in health sector spending, and expanded health system infrastructure were touted as contributors to improvements to MCH initiatives such as vaccination and ANC [49]. One review of MCH programs in 144 LMICs found that several key strategies were utilized to reduce maternal and child mortality, including good governance, multisectoral partnerships and leadership, and use of data to drive decision-making [50]. An equity-based focus has also been championed to advance progress on maternal and newborn survival via FBDs. Notably, strong political leadership along with evaluation mechanisms and space for adaptation have been identified as critical to achieving meaningful structural reforms [51]. Key implementation strategies, contextual factors, and successful EBIs in other countries that have successfully reduced U5M – along with the full report on the Peru case study from the Exemplars in U5M study – may be found on the Exemplars in Global Health website [14].

Although U5M rate globally fell from 90.6 deaths per 1,000 live births in 1990 to 42.5 deaths per 1,000 in 2015, the Millennium Development Goal of a two-thirds reduction in U5M during this period was only achieved in East Asia and the Pacific and in Latin America and the Caribbean [52]. If U5M remains stagnant at 2015 levels in each country, 94.4 million children under 5 are estimated to die between 2016–2030 [52].

Child health is expected to be further impacted by the COVID-19 pandemic; some estimates project a 45% increase in child deaths in LMICs. Understanding positive success stories from other countries – and their prioritization of child health programs during emergencies and other difficult historical moments of social and political instability – may be key to mitigating the indirect effects of COVID-19 on child health today [53].

From the results we can conclude that there are replicable lessons from Peru that would be relevant for other countries aiming to accelerate decline in U5M (Table 1). In addition to Peru, relevant lessons from other Exemplar countries may be adapted for other countries seeking to implement EBIs and successful strategies to prevent unnecessary child deaths [14].

**Table 1:** Replicable lessons from Peru that would be relevant for other countries aiming to accelerate decline in U5M

1. Embed the implementation strategies into broader efforts for equity, addressing vulnerability of the most disadvantaged through anti-poverty initiatives and plans for equity in implementation (with improved focus on the most vulnerable populations) from the start while working to improve care for all
2. Ensure national commitment to U5M reduction that is resilient to changes in government and leadership by integrating into agreements designed to be sustained with the inclusion of civil society, political parties and various components of the government – not only the MOH
3. Build research and data-driven decision-making capacity at a national level and ask for and use available evidence or develop locally relevant, culturally-sensitive evidence for decision-making to determine need and appropriateness of EBIs, identify where adaptation is needed, and what key implementation strategies are needed based on global and local factors and results during both planning and implementation
4. Integrate new initiatives into existing system capacity and promote a primary care-focused model
5. Engage the community to understand challenges before and during implementation and be willing to adapt to make it culturally appropriate and acceptable
6. Engage and consult stakeholders and leverage their expertise during planning and throughout implementation, including MOH representatives, donors, implementing partners, professional bodies, and communities

After the study period, several challenges remain in Peru’s continuing efforts to reduce U5M. Despite making significant strides to address health disparities, inequities in both coverage and outcomes still exist, specifically between regions of Peru where wealth and resource distribution vary greatly. Peru’s health system infrastructure remains fragmented, and data quality issues continue to pose a challenge to program monitoring and the use of data for decision-making [54]. These areas of improvement should remain a focus of the MOH as they continue their efforts to reduce preventable child deaths in the country.

This research included some limitations. First, due to resource constrains it was not possible to include key informants for each of the successful EBIs. Rather, the study team tried to engage several key informants with broad knowledge of many EBIs. Second, there may have been recall bias when asking key informants in 2019 to recall program strategies from 2000–2015. To allay the potential effects of recall bias, results from qualitative interviews were compared against quantitative results available in previously published literature. Third, the research methodology does not allow for the analysis of these EBIs to specifically link interventions to reductions in U5M and quantify the extent of their success in achieving the Millennium Development Goals. Rather, this work is intended to explore the implementation aspects of interventions that aim to reduce U5M. The application of a framework of implementation research to the analysis, the desk review and the use of mixed methods are important strengths that led to clear and transferable lessons.

## Conclusion

Peru has achieved a remarkable reduction in U5M due to an increase in coverage of some EBIs and contextual factors such as sustained economic growth and strong national prioritization of health initiatives. Key implementation strategies such as a focus on equity and utilizing research and data to guide healthcare decision-making and implementation contributed to successful programs. Understanding the underlying mechanisms and pathways that led to this reduction in infant and child deaths is paramount to replicating this public health success in other LMICs.

## Data Availability

Qualitative data access is restricted to users with appropriate ethics approval from the committees listed in the Ethical Considerations section. A reader or reviewer may apply to the authors for access by providing a written description of background, reasons, and intended use. If the methodology does not violate the condition of informed consent under which the interview was conducted, and the proposal approved by UGHE and other relevant ethics boards, the user can obtain the data from the corresponding author, and include one of the authors in the project and analysis.

## Appendix 1

Table 2

**Table 2:** List of infant and child evidence-based interventions

Cause of Death	EBI	
Lower respiratory infections	Antibiotic treatment	
	Vaccination: PCV	
	Vaccination: Hib	
	Community-based management	
	Facility-based management	
Diarrheal diseases	Oral rehydration therapy	
	Zinc supplementation	
	Vaccination: Rotavirus	
	Community-based management	
	Facility-based management	
Malaria	Antimalarial combination therapy	
	Rapid diagnostic testing	
	Insecticide-treated nets	
	Indoor residual spray	
	Intermittent preventative therapy for high-risk groups	
	Community-based management	
	Facility-based management	
Measles	Vaccination: Measles	
	Vitamin A supplementation (prior to vaccination)	
Malnutrition	Exclusive breastfeeding for six months	
	Continued breastfeeding and complementary feeding after six months	
	Vitamin A supplementation	
	Management of severe acute malnutrition (ready-to-use food, rehydration, antibiotics)	
HIV	ARV treatment for infants and children	
	HIV testing of children born to HIV + mothers	
	Prevention of mother-to-child transmission	Early diagnosis of pregnant women (or pre-pregnancy)
		PMTCT treatment for mothers^a^ and post-partum to exposed infants
		Elective C-section for untreated HIV + mothers**; replacement feeding^b^
		ARV treatment for mother for life as prevention (started in 2012)
		Exclusive breast feeding
Meningitis	Vaccination: PCV meningococcal	
	Vaccination: Hib	
	Vaccination: Meningococcal	
	Antibiotic treatment	
	Chemoprophylaxis during acute outbreaks	
Other vaccine preventable diseases	Vaccination: Tetanus	
	Vaccination: Diphtheria	
	Vaccination: Pertussis	
	Vaccination: Polio	

*ARV* Anti-retroviral therapy, *PCV* Pneumococcal conjugate vaccine, *PMTCT* Prevention of mother-to-child transmission of HIV.

^a^No longer recommended (PMTCT versus ART for life).

^b^No longer recommended for women on ART with suppressed viral load.

Table 3

**Table 3:** List of neonatal evidence-based interventions

Period of risk	EBI	
Preconception	Folic acid supplementation	
Antenatal	Tetanus vaccination	
	Malaria prevention and treatment	Intermittent presumptive treatment
		Insecticide-treated bed nets
	Iodine supplementation (in endemic iodine deficient settings)	
	4 or more antenatal visits (ANC4)	
	Prevention and treatment of preeclampsia and eclampsia	Antihypertensive treatment for severe hypertension
		Magnesium sulfate
		Early delivery
Intrapartum	Antibiotics for preterm premature rupture of membranes	
	Corticosteroids for preterm labor	
	C-section for breech or obstructed labor	
	Active management of delivery (including partograph)	
	Clean delivery practices (incl. clean cord-cutting)	
	Trained birth attendant	
	Facility-based delivery	
	Basic emergency obstetric and newborn care (BEmONC)	
	Comprehensive emergency obstetric and newborn care (CEmONC)	
	Timely transport for higher level care for mother	
Postnatal	Newborn resuscitation	
	Immediate breastfeeding	
	Prevention and management of hypothermia	Immediate drying and wrapping
		Delayed bathing
		Skin-to-skin
		Baby warming
	Kangaroo care for LBW/prematurity	
	Timely transport for higher level care for mother	
	Post-partum visits to identify danger signs and provide active referral	
	Antibiotics for suspected or confirmed infection	
	Surfactant therapy for respiratory distress syndrome and prematurity	
	Neonatal intensive care units (equipped, trained staff, standards and protocols established and followed)	

## Abbreviations

AN: Acuerdo Nacional (National Agreement)
ANC: Antenatal care
ANC4: Four or more antenatal care visits
DHS: Demographic and Health Survey
EBI: Evidence-based intervention
EPIAS: Exploration, Preparation, Implementation, Adaptation, and Sustainability
FBDs: Facility-based deliveries
Hib: Hemophilus influenza b vaccine
LMICs: Low- and middle-income countries
MCH: Maternal and child health
MCLCP: Mesa de concertación para la lucha contra la pobreza (Roundtable for the Fight Against Poverty)
MOH: Ministry of Health
NM: Neonatal mortality
PCV: Pneumococcal conjugate vaccine
SIS: Seguro Integral de Salud (Comprehensive National Health Insurance System)
U5M: Under-5 mortality
